# A STUDY ON COOPERATION BETWEEN COMMUNITY COMPREHENSIVE SUPPORT CENTERS AND DV SUPPORT AGENCIES TO SOLVE ELDER ABUSE

**DOI:** 10.1093/geroni/igad104.2904

**Published:** 2023-12-21

**Authors:** Asako Katsumata, Noriko Tsukada

**Affiliations:** Japan Lutheran College Graduate School of Social Work, Chiba, Tokyo, Japan; Nihon University, Setagata-city, Tokyo, Japan

## Abstract

The purpose of this study was to examine current and future cooperation between Community Comprehensive Support Centers (CCSCs) and Domestic Violence (DV) support agencies in dealing with elder abuse cases among older couples in Japan. The data used in this study are from a nationwide survey from Certified Social Workers (CSWs) at CCSCs in November 2022. A structured questionnaire survey was provided to a randomly selected 3,563 out of 5400 CCSCs, and a total of 1,472 responses from CSWs were obtained (response rate of 41.3%). Preliminary analyses revealed about 70% of the respondents felt that the cooperation with DV support agencies was important (36.8% “important”, 32.6% “somewhat important“). Reasons for why respondents felt the cooperation was “not very important” (2.2%), “not important at all”(0.1%), or “neutral”(28.3%) included “have never cooperated with DV support agencies before” (62.1%), “CCSCs are supposed to deal with abuse cases with people aged more than 65“ (55%), “don’t know about roles of DV support agencies” (42.5%) , followed by “staff members at DV support agencies were not very helpful” (28.1%). Moreover, among 443 respondents (30.1%) who have dealt with elder abuse cases that have continued from their younger age, 25% of CSWs sought for cooperation with DV support agencies, got help and shared information to solve those cases. Findings suggest that professional staff members at both CCSCs and DV support agencies need to be trained to be aware of importance of exchanging their knowledge and resources regarding how to solve elder abuse cases effectively and efficiently.

